# Identifying High-Risk Spatiotemporal Clusters of Mushroom Poisoning in Subtropical China: A Retrospective Surveillance Study in Zhejiang Province (2012–2023)

**DOI:** 10.3390/foods15162913

**Published:** 2026-08-20

**Authors:** Sitong Xu, Haoyi Zhang, Lili Chen, Lei Fang, Haizhu Jiang, Ronghua Zhang, Jiang Chen, Hexiang Zhang, Xiaojuan Qi, Yue He, Bing Zhu, Jikai Wang, Ting Liu

**Affiliations:** 1 Institute of Remote Sensing and Earth Sciences, Hangzhou Normal University, Hangzhou 311121, China; 2023210214058@stu.hznu.edu.cn (S.X.); 2023210214060@stu.hznu.edu.cn (H.J.); 2 School of Public Health, Xiamen University, Xiamen 361005, China; zhyxmu3475@stu.xmu.edu.cn; 3 Department of Nutrition and Food Safety, Zhejiang Provincial Center for Disease Control and Prevention, 3399 Binsheng Road, Binjiang District, Hangzhou 310051, China; llchen@cdc.zj.cn (L.C.); rhzhang@cdc.zj.cn (R.Z.); jchen@cdc.zj.cn (J.C.); hxzhang@cdc.zj.cn (H.Z.); xjqi@cdc.zj.cn (X.Q.); yhe@cdc.zj.cn (Y.H.); bzhu@cdc.zj.cn (B.Z.); 4 Department of Environmental Science and Engineering, Fudan University, Shanghai 200438, China; fanglei@fudan.edu.cn

**Keywords:** mushroom poisoning, epidemiology, spatial statistics

## Abstract

To understand the epidemiological characteristics and patterns of mushroom poisoning in Zhejiang Province from 2012 to 2023, and to overcome the limitations of previous descriptive studies in precise early warning and spatial identification, this study explored the feasibility of identifying spatial distribution characteristics and high-risk spatiotemporal clusters. First, descriptive epidemiological analysis was conducted on 2276 cases from the Foodborne Disease Case Surveillance System and 408 outbreaks from the Foodborne Disease Outbreak Surveillance System reported over the 12-year period to clarify the basic characteristics and trends of poisoning. Subsequently, spatial autocorrelation analysis (Moran’s *I*) was employed to reveal spatial dependence and clustering patterns. Finally, spatiotemporal scan statistics (SatScan) were used to precisely identify high-risk spatiotemporal clusters, systematically analyzing the spatiotemporal distribution and clustering patterns of mushroom poisoning cases. The results showed a distinct summer–autumn seasonal peak (June–October), attributed to the subtropical monsoon climate with high temperatures and abundant rainfall, which is conducive to mushroom growth. Farmers were the most affected population (47.93%), and homes were the primary poisoning locations (71.7%), reflecting widespread foraging habits and insufficient risk awareness in rural areas. *Chlorophyllum molybdites* (36.27%) and *Russula japonica* (10.05%) were the dominant poisoning mushroom species, with gastrointestinal symptoms being the predominant clinical manifestation (84.07%). Spatial analysis revealed significant spatiotemporal clustering of mushroom poisoning in Zhejiang Province. The global Moran’s *I* index showed significant positive autocorrelation in some years (*p* < 0.05), with local hotspots mainly distributed in western Zhejiang counties. This pattern is driven by a dual model of environmental suitability and behavioral risk, resulting from the high forest coverage and humid climate of the western Zhejiang mountainous areas providing suitable habitats, combined with long-standing foraging habits among local residents. Retrospective spatiotemporal scanning identified high-risk clusters for each year from 2018 to 2023, with the Lishui area in 2023 being the most significant cluster (Relative Risk (RR) = 15.44, Log-Likelihood Ratio (LLR) = 114.49). The results confirm that mushroom poisoning in Zhejiang Province exhibits a stable and identifiable spatiotemporal clustering pattern, providing a quantitative basis for precise health education and targeted prevention and control in high-risk counties of western Zhejiang during June–October, thereby shifting the approach from passive reporting to targeted intervention.

## 1. Introduction

Mushroom poisoning is a prominent public health issue in China, characterized by strong regionality, concentrated occurrence periods, and household clustering. Recent studies indicate that China, Russia, and Eastern European countries have among the highest numbers of mushroom poisoning cases and fatality rates globally [1,2]. In 2023, China reported a total of 6960 foodborne disease outbreaks [3]. Mushroom poisoning events were the most frequent, accounting for 44.35% of all outbreaks, and represent one of the leading causes of death in food poisoning incidents [3].

Mushrooms refer to a group of large fungi with fleshy to nearly fleshy fruiting bodies. Globally, approximately 14,000 species of mushrooms are currently known, with an estimated 4000 species identified in China. Among these, 936 species have edible value [4], and 437 species have medicinal value [5]. Poisonous mushrooms, also known as toxic mushrooms or poisonous fungi, refer to species whose fruiting bodies cause poisoning reactions in humans, livestock, or poultry upon consumption [6]. Among the mushroom species in China, 435 are poisonous, spanning 5 classes, 14 orders, 49 families, and 126 genera [7], with over 30 species being highly toxic and potentially lethal, and at least 16 species being extremely toxic [8]. Due to their high nutritional value, delicious taste, and minimal contamination from pesticides, wild mushrooms are widely cherished as organic food by people around the world. A small number of people consume wild mushrooms for recreational hallucinogenic purposes [9]. However, the diversity of wild mushrooms, coupled with the fact that non-toxic and toxic species often grow together, leads to frequent poisoning incidents worldwide each year due to the mistaken collection and consumption of toxic mushrooms [10,11,12]. If patients with toxic mushroom poisoning are not treated promptly, it can result in death, directly endangering public health and life. Therefore, poisoning from the accidental ingestion of toxic mushrooms is consistently regarded as a global threat to human health. China has a long history of collecting and consuming wild mushrooms, which are traditionally believed to have higher nutritional and medicinal value, resulting in a large number of toxic mushroom poisoning cases or incidents each year.

Zhejiang Province, located on the southeast coast of China, has high forest coverage and a humid climate suitable for mushroom growth. In some areas, residents or migrant workers have the habit of foraging for wild mushrooms. Although relevant government departments issue public education and early warning information about mushroom poisoning annually, the overall number of sporadic cases and outbreak incidents of mushroom poisoning has shown an increasing trend [13]. Current analysis of surveillance data mostly relies on traditional descriptive epidemiology of person, place, and time, representing a passive reporting model that makes it difficult to identify early case clustering associations or clarify poisoning patterns and major influencing factors, resulting in a lack of timeliness and precision in early warning efforts. Classical statistical analyses often ignore spatial autocorrelation, making it difficult to objectively and quantitatively assess disease clustering over large areas, and they are prone to introducing selection bias. Mushroom poisoning has a distinct geographical nature. The use of spatial statistical methods can more fully incorporate geographic spatial background information, making research results more scientific and objective. These methods have been widely applied in the field of public health for disease cluster analysis, risk factor exploration, and spatiotemporal pattern prediction [14,15].

The value of spatiotemporal statistical methods in disease surveillance has been confirmed by multiple studies. In the field of infectious diseases, Lan Li et al. [16] used spatial statistics, spatial autocorrelation analysis, and spatiotemporal scan statistics to reveal significant spatiotemporal clustering characteristics of tuberculosis in Beijing from 2009 to 2014, providing a basis for targeted interventions in high-risk areas. Suleman Atique et al. [17] identified spatiotemporal clustering of dengue fever in the Swat region through spatiotemporal scanning, with findings contributing to future epidemic response. Hua Gu et al. [18] applied spatial statistical methods to analyze typhoid and paratyphoid fever cases in Zhejiang Province, showing different clustering patterns and risk factors for the two diseases. Lukáš Marek et al. [19] used spatiotemporal scan statistics to identify potential links and spatial distribution of campylobacteriosis, suggesting the possible role of environmental exposure factors. In the field of foodborne diseases, the spatiotemporal scan statistics method has also demonstrated good application potential: Pearl et al. [20] applied it to outbreak detection of *Escherichia coli* O157:H7; So et al. [21] combined molecular typing information to identify spatiotemporal clustering of cases with specific PFGE types; Varga et al. [22] applied it to the analysis of *Salmonella* Enteritidis infections with different phage types, preliminarily confirming the applicability of this method in foodborne disease outbreak surveillance. Most of the aforementioned studies focus on pathogens with interpersonal transmission characteristics, such as bacterial and viral agents, or rely on clear laboratory typing information for cluster detection. In contrast, mushroom poisoning exhibits significantly different epidemiological characteristics: its pathogenic agents are naturally occurring fungal toxins in the environment, with cases entirely resulting from environmental exposure and individual foraging behavior, lacking interpersonal transmission attributes. Its spatiotemporal clustering is not only driven by natural factors such as climate and vegetation but also deeply coupled with social behavioral factors such as local dietary culture and risk perception levels. This exposure model, driven by both environmental and human behavioral factors, makes the spatiotemporal clustering mechanism of mushroom poisoning more complex than that of traditional infectious diseases, making it difficult to directly adapt existing models.

Accordingly, the value of this study lies not in simply transplanting spatiotemporal statistical tools into the field of poisonous mushroom poisoning, but in making substantive advancements in the following three aspects in response to the aforementioned particularities.

First, unlike previous studies in Zhejiang Province that were short-term or geographically restricted to a small number of districts and counties, this study draws on 12 years of complete surveillance data covering 90 districts and counties across the province to present an unprecedented comprehensive spatiotemporal landscape for this region.

Second, in addition to employing Moran’s *I* and SaTScan for conventional spatial cluster detection, this study systematically evaluates the potential impact of the surveillance system’s expansion during the study period—with the number of reporting institutions growing from 20 to 1785—on the observed spatiotemporal trends, and incorporates this factor as a key explanatory variable into the analytical framework. This consideration has rarely been addressed in prior similar studies.

Third, this study distinguishes the niche characteristics of two dominant poisonous mushroom species in Zhejiang Province: *Chlorophyllum molybdites*, a saprotrophic fungus in anthropogenic habitats, and *Russula japonica* sensu lato, a symbiotic fungus in forest ecosystems. It proposes region-specific and season-specific prevention and control strategies that are markedly distinct from those in studies in southwest China, which center on the highly lethal genus *Amanita*.

Based on the foodborne disease surveillance network of Zhejiang Province from 2012 to 2023, this study comprehensively employs spatiotemporal statistical methods including descriptive epidemiology, spatial autocorrelation analysis, and spatiotemporal scan statistics. Through retrospective analysis, it addresses the following three objectives: (1) transforming the high incidence of mushroom poisoning from qualitative descriptions into quantitative indicators, precisely locating high-risk spatiotemporal clusters to specific districts/counties and date windows; (2) explaining why high-risk clusters form by combining population distribution, environmental characteristics, and behavioral factors; and (3) proposing specific strategies for precision prevention and control based on the identified spatiotemporal clustering results. Through the above analysis, this study systematically validates the applicability of retrospective spatiotemporal statistical methods in mushroom poisoning surveillance, providing a methodological foundation and empirical evidence for the transition from passive reporting to targeted intervention.

## 2. Materials and Methods

### 2.1. Data Sources

**Figure 1:**
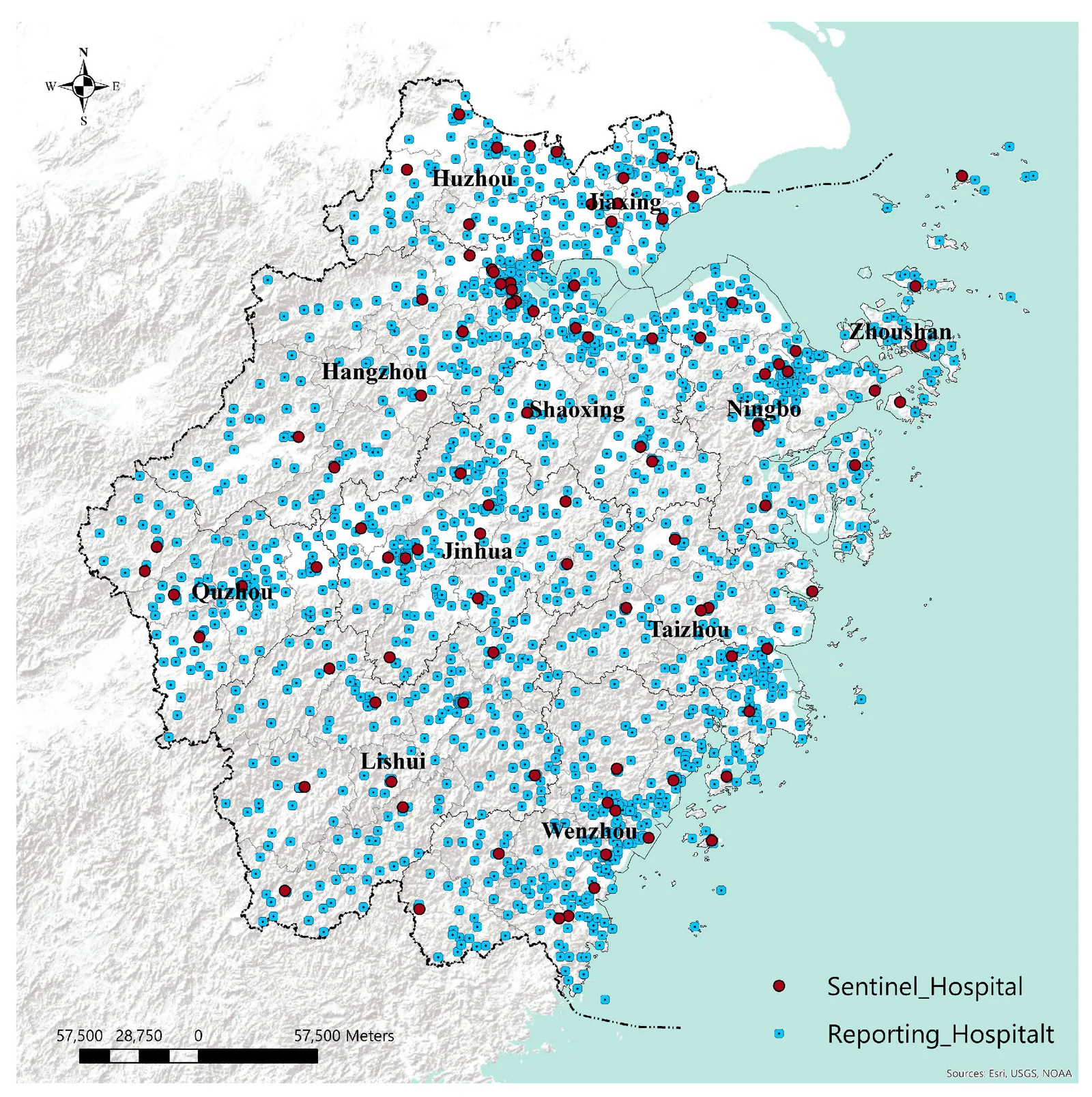
Distribution map of medical institutions in Zhejiang Province. Red dots represent sentinel posts, and blue dots represent reporting posts.

The study selected Zhejiang Province as the research area, including 90 districts and counties across 11 prefecture-level cities such as Hangzhou, Ningbo, and Wenzhou (as shown in Figure 1). The data were sourced from the Zhejiang Province Foodborne Disease Surveillance Reporting System, which includes case-based surveillance and outbreak surveillance records from January 2012 to December 2023. For species identification, all mushrooms with definitive Latin scientific names or Chinese species names were confirmed by professional mycologists based on macroscopic and microscopic morphological characteristics. For cases without clear mushroom species information, the causative species was comprehensively inferred via field epidemiological investigations, patients’ mushroom consumption history and consistent clinical manifestations. No DNA molecular identification or sequencing validation was performed in this retrospective surveillance dataset.

During the study period, the provincial surveillance network continued to expand. From 2012 to 2015, the number of medical institutions participating in surveillance increased from 10 to 101. Since 2016, all secondary-level and above medical institutions at or above the county level across the province have been fully incorporated into the foodborne disease surveillance system. In 2018, Zhejiang Province launched a province-wide special surveillance program for wild mushroom poisoning, further extending the surveillance network to cover all medical institutions receiving and treating patients with foodborne diseases, including community health service centers and township health centers. As of 2023, the surveillance network covered approximately 1790 medical institutions.

The address information of each reporting medical institution was converted into geospatial coordinate data via the Baidu Maps Geocoding API (https://lbsyun.baidu.com/apiconsole/key, accessed on 20 May 2026).

Population data for each city and district/county from 2012 to 2023 were obtained from the Zhejiang Provincial Bureau of Statistics (https://tjj.zj.gov.cn/col/col1525563/index.html, accessed on 20 May 2026), and GIS map data for Zhejiang Province were sourced from the National Geomatics Center of China (https://www.ngcc.cn/, accessed on 20 May 2024).

Multiple ecological background indicators were extracted in this study to conduct an exploratory analysis of the environmental context.

The Normalized Difference Vegetation Index (NDVI) data were obtained from the Resource and Environment Science and Data Center (https://www.resdc.cn/, accessed on 28 April 2021), and were used to characterize regional vegetation greenness. Based on NDVI data covering the high-incidence season of mushroom poisoning (June to October) from 2018 to 2023, the mean high-incidence-season NDVI for each county-level unit was calculated annually, and the multi-year average was further derived as the county-level vegetation indicator.

Forest cover proportion data were sourced from the ESA WorldCover v200 global land cover dataset released by the European Space Agency. Generated from annual remote sensing imagery, this dataset has a spatial resolution of 10 m. In this study, raster pixels corresponding to the tree cover type were extracted, and the proportion of tree-covered land area within each county-level administrative unit was calculated to quantify county-level forest coverage.

Regional elevation data were derived from the SRTMGL1_003 dataset of the United States Geological Survey (USGS), with a spatial resolution of 30 m. The average elevation of each county was calculated based on elevation rasters within its administrative boundary.

The above ecological indicators are only used to describe the ecological background characteristics of recurrent clustering areas, and are not intended to infer causal relationships between environmental factors and poisonous mushroom poisoning.

### 2.2. Research Framework and Methods

**Figure 2:**
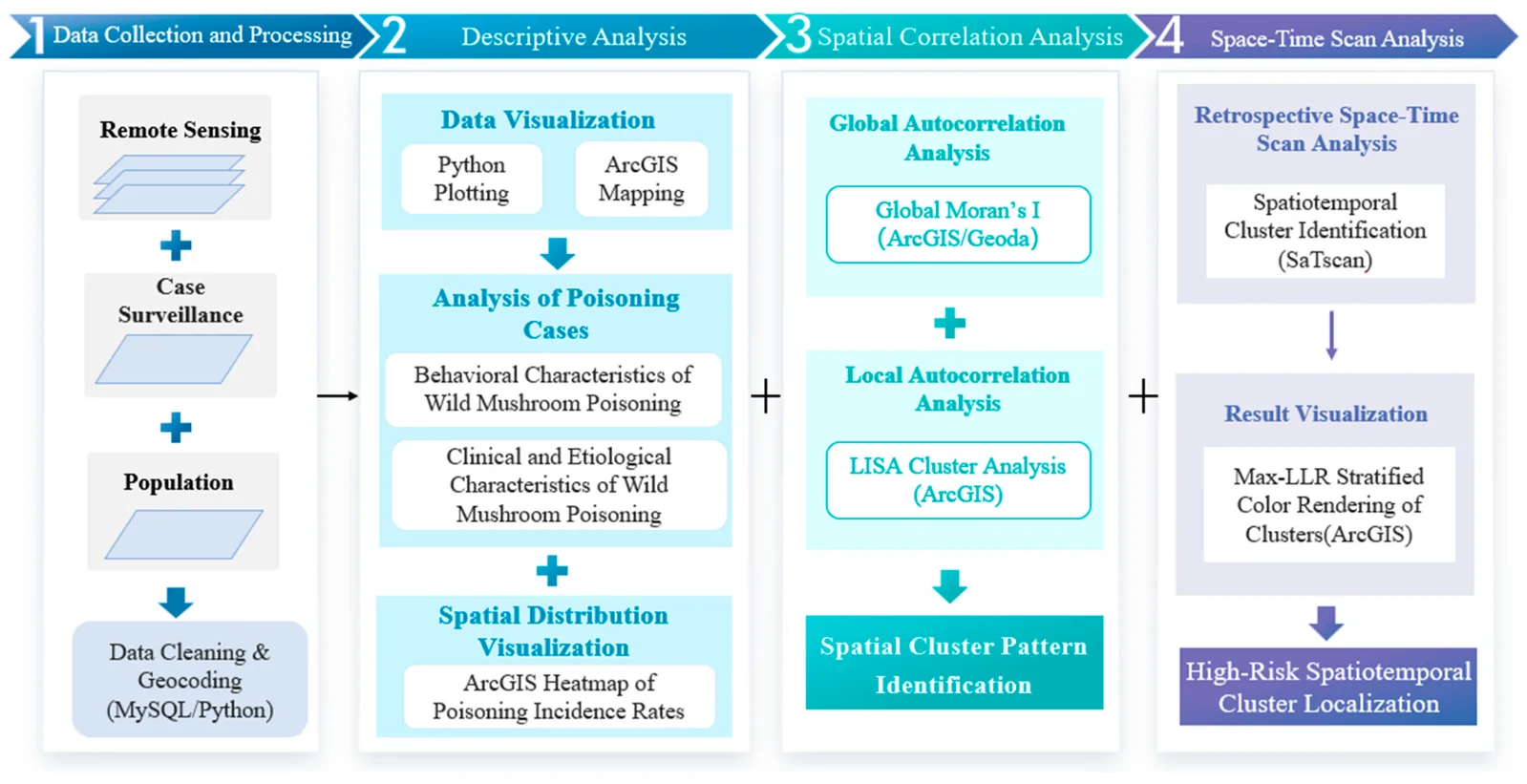
Research framework.

This study adopts a four-stage progressive process of “Data Preprocessing—Descriptive Analysis—Spatial Correlation Analysis—Spatiotemporal Scanning,” integrating multi-source data to construct a unified spatiotemporal database (Figure 2). By utilizing Python 3.12, ArcGIS 10.8, GeoDa 1.20, and SaTScan v10.3.3 software, it systematically reveals the spatiotemporal distribution characteristics of wild mushroom poisoning in Zhejiang Province, identifies high-risk spatiotemporal clusters, and provides a scientific basis for precise prevention and control.

To characterize long-term epidemiological patterns while detecting annual-scale spatiotemporal clusters, this study adopted an analytical strategy based on stratified time windows:

(1) Analysis of long-term epidemiological characteristics (2012–2023). Descriptive epidemiological analysis was performed on case data covering the full study period to systematically depict the temporal trends, population characteristics, and case distribution patterns of poisonous mushroom poisoning in Zhejiang Province.

(2) Spatiotemporal cluster analysis after the launch of special surveillance (2018–2023). Given that Zhejiang Province launched a special surveillance program for poisonous mushroom poisoning in 2018 and the coverage of its foodborne disease surveillance network was further expanded, the 2018–2023 period was selected for annual spatial autocorrelation analysis and retrospective spatiotemporal scan analysis so as to identify spatial clustering patterns and high-risk spatiotemporal clusters across different years. Furthermore, cluster recurrence frequency analysis was conducted based on the annual spatiotemporal scan results to identify persistent high-risk areas.

#### 2.2.1. Descriptive Epidemiological Analysis

A case database (from the Foodborne Disease Case Surveillance System) was established using MySQL 8.0 for descriptive analysis. Python 3.12 was used to visualize case data including time, occupation, and age, while ArcGIS10.8 was employed to visualize the spatial distribution of poisonous mushroom incidence rates. Reported poisoning incidence rate (%) = (Annual reported case count/local permanent resident population) × 100.

#### 2.2.2. Spatial Autocorrelation Analysis

A spatial autocorrelation model was used to analyze the spatial clustering of mushroom poisoning cases. Spatial autocorrelation refers to the correlation of the same variable across different spatial locations and serves as a measure of the degree of clustering of attribute values in spatial units. This study employs the Moran’s *I* index from global spatial autocorrelation analysis and the Moran’s *I* index from local spatial autocorrelation analysis as indicators for spatial autocorrelation analysis. The statistical significance of Moran’s *I* was tested using Monte Carlo simulation with 999 random permutations. For each study year, the observed Moran’s *I* was compared against the reference distribution generated by randomly reallocating incidence rates across all spatial units. The *p*-value was defined as the proportion of permutations yielding a Moran’s *I* value greater than or equal to the observed value (i.e., as extreme as the observed statistic). A *p*-value of less than 0.05 was considered statistically significant.

(1) Global Spatial Autocorrelation

Global spatial autocorrelation describes the spatial characteristics of related attribute values across the entire region, thereby testing whether clustering effects exist within the whole region. Among these, Moran’s *I* is an important indicator for analyzing clustering effects, and its calculation formula is as follows:

$$I=\frac{n}{S_{0}}\frac{\sum_{i=1}^{n}\sum_{j=1}^{n}\omega_{ij}\left(x_{i}-\bar{x}\right)\left(x_{j}-\bar{x}\right)}{\sum_{i=1}^{n}\sum_{j=1}^{n}\omega_{ij}\sum_{i=1}^{n}{\left(x_{i}-\bar{x}\right)}^{2}}$$

In the formula, *n* represents the number of counties (districts) in Zhejiang Province. and denote different counties (districts). x_i_ and x_j_ are the observed values at spatial locations i and j, and ω_ij_ represents the adjacency relationship between spatial locations i and j. When i and j are adjacent spatial locations, ω_ij_ = 1; otherwise, ω_ij_ = 0. The value of the global Moran’s *I* ranges from [−1, 1]. When Moran’s *I* > 0 and *p* < 0.05, it indicates the presence of high-value or low-value clusters of cases; when Moran’s *I* < 0 and *p* < 0.05, it indicates a dispersed distribution of cases; when Moran’s *I* = 0 and *p* ≥ 0.05, it indicates no clustering of cases, suggesting a random distribution. Global spatial autocorrelation analysis uses the Queen Contiguity method to create a spatial weight matrix, conducting a year-by-year global autocorrelation analysis of the incidence rate of poisonous mushrooms at the city level in Zhejiang Province.

(2) Local Spatial Autocorrelation

Local spatial autocorrelation is used to detect local spatial heterogeneity and identify clustering or dispersion patterns that may be masked by global analysis. This study employs the local Moran’s *I* index proposed by Anselin (1995) [23], with the calculation formula as follows:

$$I_{i}=\frac{\left(x_{i}-\bar{x}\right)}{S^{2}}\sum_{j}C_{ij}\left(x_{j}-\bar{x}\right)$$

where xi is the reported incidence rate of poisonous mushroom poisoning in the county, $\bar{x}$ is the average incidence rate across all counties in the province, S^2^ is the variance, and C_ij_ is the element of the spatial weight matrix, representing the spatial adjacency relationship between counties i and j (using the Queen Contiguity method, where C_ij_ =1 if adjacent, and 0 otherwise). If the local Moran’s *I*_i_ is positive, it indicates that the incidence rate in the county and its neighboring counties exhibits clustering in the same direction, meaning high-incidence areas are surrounded by high-incidence areas (high–high clustering, i.e., “hot spots”) or low-incidence areas are surrounded by low-incidence areas (low–low clustering, i.e., “cold spots”). If I_i_ is negative, it indicates spatial heterogeneity, meaning high-incidence areas are surrounded by low-incidence areas (high–low dispersion) or low-incidence areas are surrounded by high-incidence areas (low–high dispersion). The incidence patterns of 90 counties in Zhejiang Province are visualized using a Local Indicators of Spatial Association (LISA) cluster map.

Based on cumulative data from 2012–2023, this study conducts local spatial autocorrelation analysis at the county level as the spatial unit, aiming to identify long-term stable local hot spots and cold spots.

#### 2.2.3. Spatiotemporal Cluster Analysis

Spatial autocorrelation analysis revealed the spatial clustering pattern of poisonings. To simultaneously capture spatiotemporal interactions and quantify clustering risk, the time dimension was introduced. This study applied the spatiotemporal scan statistic method to analyze the spatiotemporal clustering of mushroom poisoning cases. This method is based on a moving scanning window that integrates both temporal and spatial information, making it suitable for identifying abnormal clusters of foodborne diseases within specific regions and time periods. The basic approach involves constructing a two-dimensional cylindrical spatiotemporal window, where the base of the cylinder represents the geographic range and the height corresponds to the time span. By dynamically adjusting the geographic radius and time length of the window, the entire study area and period are scanned to detect potential cluster regions.

During the analysis, it is assumed that the number of cases follows a Poisson distribution. The Log-Likelihood Ratio (LLR) is used as an indicator to measure the degree of abnormality in disease incidence within the window. The calculation formula is as follows:

$$LLR=log\left[{\left(\frac{c_{A}}{\mu_{A}}\right)}^{c_{A}}{\left(\frac{C-c_{A}}{C-\mu_{A}}\right)}^{\left(C-c_{A}\right)}\right]$$

where C is the total number of cases, c_A_ is the actual number of cases within the window, and μ_A_ is the expected number of cases within the window. In the analysis process, the number of cases is first randomly assigned to each district/county based on the population at risk. A null distribution is then constructed through multiple Monte Carlo simulations (e.g., 999 simulations) to identify the window with the largest LLR, referred to as the “most likely cluster.” Additionally, other windows with relatively high LLR values are evaluated as “secondary clusters.”

This study employed a retrospective spatiotemporal scanning analysis, scanning mushroom poisoning case data from 2018 to 2023 annually at the district/county level. Based on complete historical data, statistically significant outbreak clusters in terms of both area and time period were identified. Evaluation indicators included LLR, Relative Risk (RR), and *p*-value, where RR represents the multiple of disease risk within the cluster area compared to outside the area, and the *p*-value was used to determine whether the cluster was caused by random factors (*p* < 0.05 was considered significant).

The analysis utilized SaTScan v10.3.3 software, setting the maximum spatial cluster size to 10% of the total population, with daily-based time windows. The minimum temporal cluster size was set to 2 days and the maximum temporal cluster size to 30 days, and 999 Monte Carlo replications were performed. For cluster areas with a *p*-value less than 0.05, ArcGIS 10.2 was further employed for visualization to present the spatiotemporal clustering characteristics of mushroom poisoning cases.

To preliminarily explore the potential contribution of environmental factors to the spatial clustering of poisonous mushroom poisoning from an ecological perspective, this study further calculated the cumulative frequency with which each district and county was identified as a clustering area (including the most likely cluster and secondary clusters) in each annual spatiotemporal scan from 2018 to 2023, based on the clustering areas detected by the above methods. On this basis, all districts and counties were divided into three groups according to their cumulative frequency: the low-frequency group (≤1 time), the medium-frequency group (2–3 times), and the high-frequency group (≥4 times). Differences in the distributions of the Normalized Difference Vegetation Index (NDVI), forest coverage rate, and average elevation were compared across the three groups. The above comparison is intended to describe the general environmental characteristics of districts and counties with different clustering frequencies, rather than to establish causal relationships.

## 3. Results

### 3.1. Descriptive Analysis

#### 3.1.1. Results of Mushroom Poisoning Case Analysis

**Figure 3:**
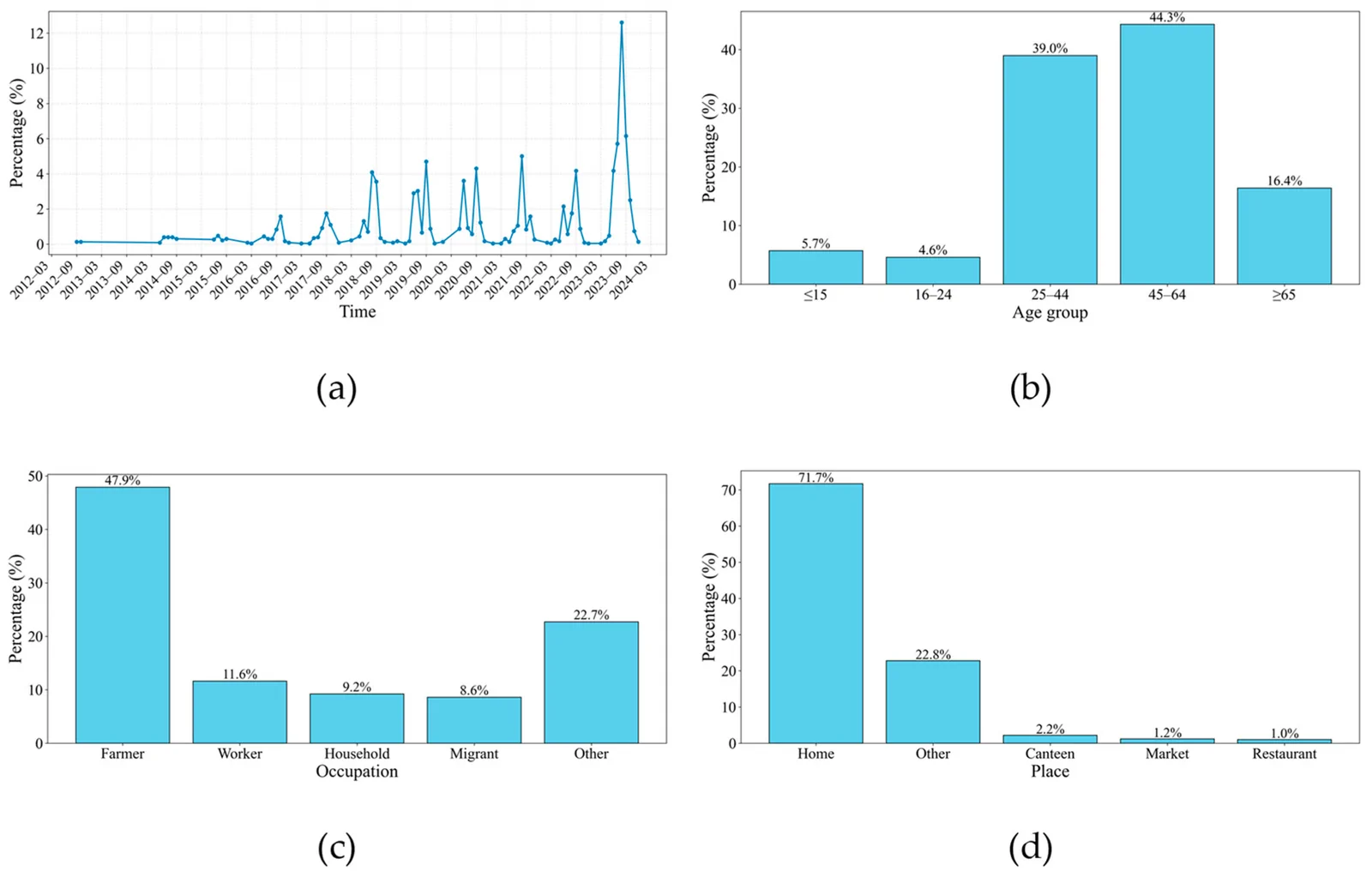
Behavioral characteristics of mushroom poisoning in Zhejiang Province, 2012–2023: (**a**) temporal distribution of mushroom poisoning cases; (**b**) age and sex distribution of mushroom poisoning cases; (**c**) occupational distribution of mushroom poisoning cases; (**d**) distribution of poisoning locations of mushroom poisoning cases.

From 2012 to 2023, a total of 2276 cases of mushroom poisoning (from the Foodborne Disease Case Surveillance System) and 408 outbreak events (from the Foodborne Disease Outbreak Surveillance System) were reported in Zhejiang Province. The temporal distribution indicates a strong seasonal pattern, with 93.8% of cases concentrated between June and October each year, peaking in August (612 cases, 26.9%) and September (605 cases, 26.6%) (Figure 3a). This stable summer–autumn peak aligns closely with the natural conditions of high temperatures and abundant rainfall under Zhejiang’s subtropical monsoon climate, which are conducive to mushroom growth, suggesting that climatic factors are a key background driver of the seasonal fluctuation in poisoning incidents.

In terms of population distribution, the age of cases ranged from 1 to 93 years, with the 30–60 age group (prime working age) predominating (67.8%) (Figure 3b). Males slightly outnumbered females (52.6% vs. 47.5%). Regarding occupational distribution, farmers accounted for the highest proportion (47.9%), followed by workers (11.6%), homemakers and unemployed individuals (9.2%), and migrant laborers (8.6%) (Figure 3c). Cases involving farmers, as the most affected group, are closely associated with their frequent exposure to wild mushrooms, common foraging and consumption habits, and limited risk identification capabilities, highlighting the central role of behavioral factors in poisoning occurrences.

Regarding the distribution of poisoning locations, households accounted for 71.7% (1632 cases), followed by other locations (22.8%), collective canteens (2.2%), and retail markets (1.2%) (Figure 3d). Households, as the primary poisoning location, further confirm the dominant role of the foraging and consumption behavior pattern, suggesting that prevention and control communication needs to penetrate rural household units.

**Figure 4:**
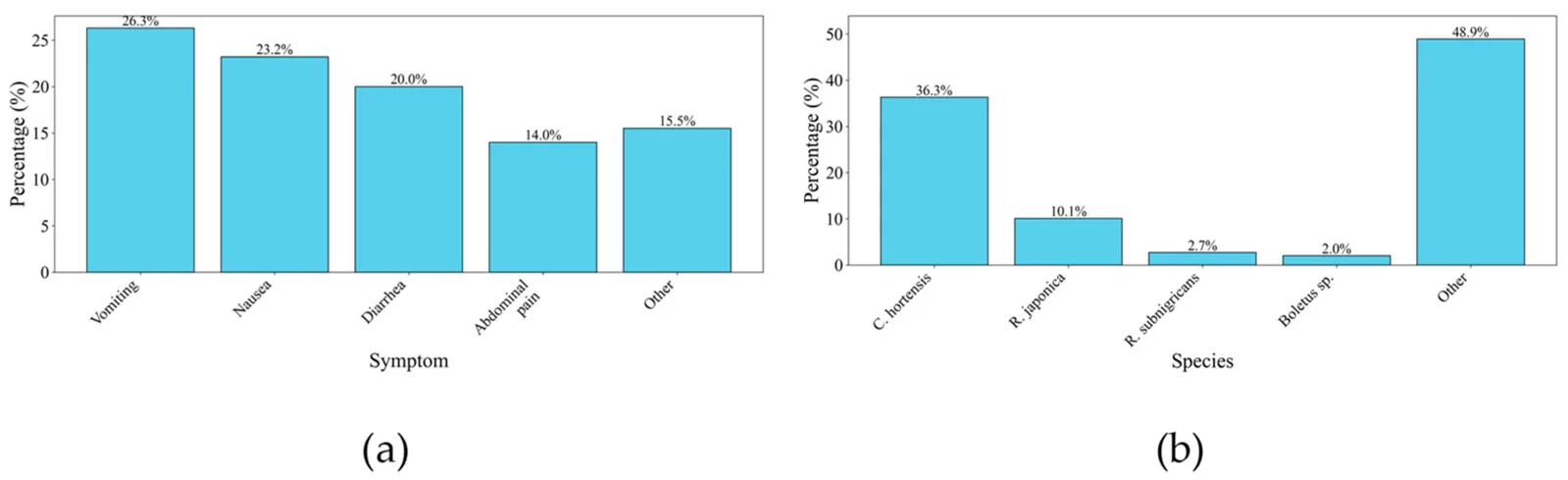
Clinical and etiological characteristics of mushroom poisoning in Zhejiang Province, 2012–2023: (**a**) distribution of clinical symptoms in mushroom poisoning cases; (**b**) distribution of poisonous mushroom species in mushroom poisoning cases.

Clinical symptom statistics indicate that acute gastroenteritis symptoms, including vomiting (26.0%), nausea (23.2%), diarrhea (20.0%), and abdominal pain (14.0%), are overwhelmingly dominant (Figure 4a). This is directly related to the toxin type (gastroenteritis type) of the dominant species in the province, *Chlorophyllum molybdites* and *Russula japonica*, and explains why, despite the low risk of fatal poisoning, the medical burden persists.

The identification results of poisonous mushroom species show that among the 408 incidents, *Chlorophyllum molybdites* was the primary causative species (148 incidents, 36.3%), followed by *Russula japonica* (41 incidents, 10.1%) (Figure 4b). Both are saprophytic or symbiotic species that thrive in areas with frequent human activity. Their dominant status reveals the ecological basis of mushroom poisoning in Zhejiang Province—namely, a high degree of overlap between species distribution and rural human living environments. Although highly toxic species such as *Russula subnigricans* and *Amanita oberwinklerana* were involved in fewer incidents, they pose a high risk of fatality (causing a total of 6 deaths), warranting vigilance against their potential threat.

#### 3.1.2. Spatial Distribution Characteristics of High Incidence

**Figure 5:**
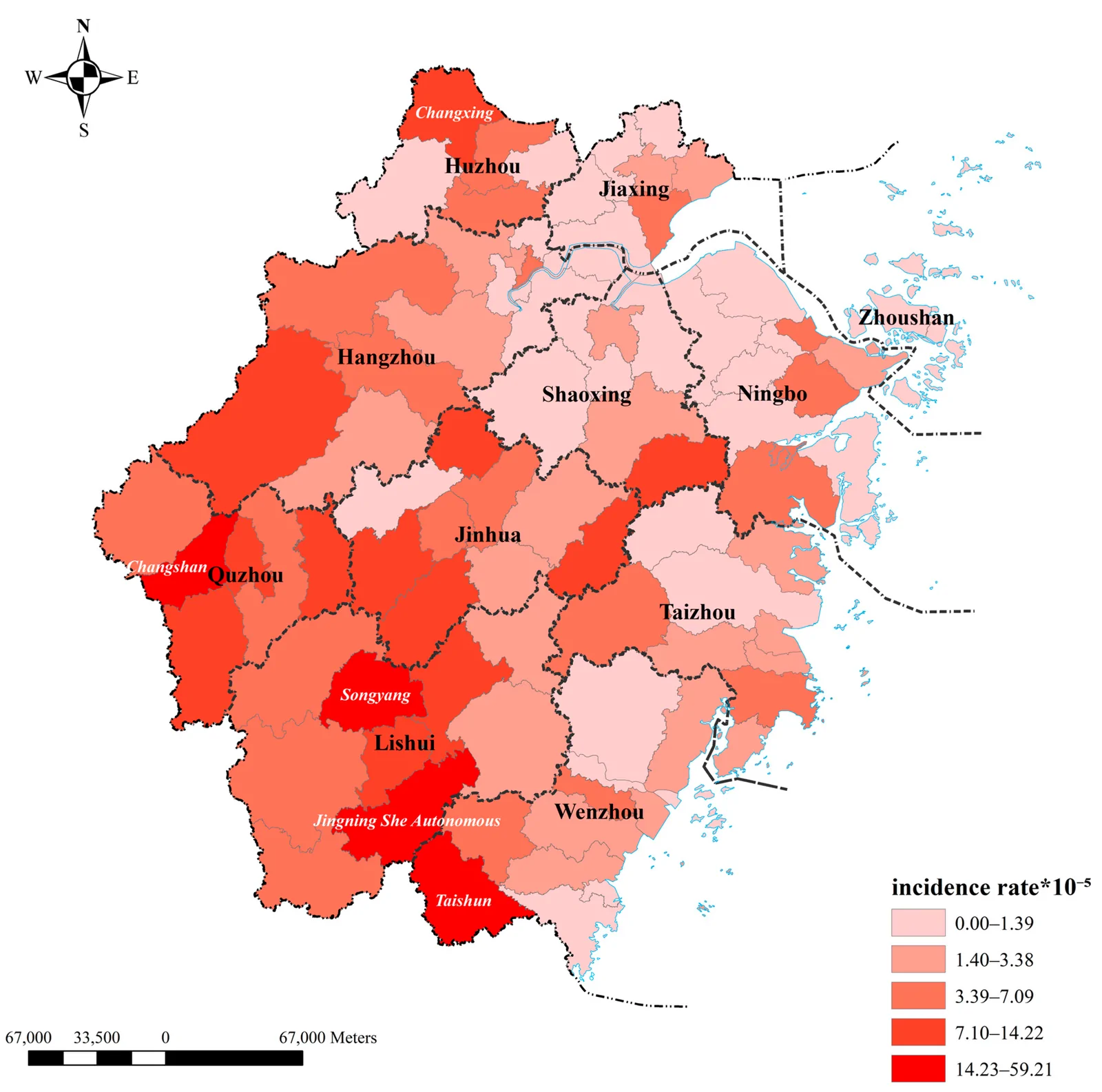
Distribution of Average Reported Incidence of Mushroom Poisoning by County in Zhejiang Province (2012–2023).

A total of 2276 cases of mushroom poisoning were reported in Zhejiang Province from 2012 to 2023. Among them, the number of mushroom poisoning cases in Zhejiang Province was relatively low from 2012 to 2015, mainly distributed in Quzhou, Lishui, Shaoxing, and Jinhua (Table 1 and Figure 5). However, since 2016, the number of cases has increased significantly, especially between 2018 and 2023, when the number of cases rose substantially. Overall, mushroom poisoning cases in Zhejiang Province have shown an increasing trend and are notably unevenly distributed.

### 3.2. Spatial Autocorrelation Analysis

Spatial autocorrelation was assessed at two spatial levels. Annual global Moran’s *I* was calculated at the prefecture-city level to evaluate broad spatial autocorrelation patterns over time, whereas local Moran’s I was conducted at the county level to identify more specific local hotspots and cold spots.

#### 3.2.1. Annual Global Moran’s *I* at the Prefecture Level

The results of the global autocorrelation analysis are shown in Table 2. In 2020 and 2021, the reported incidence rates of mushroom poisoning exhibited positive spatial autocorrelation (Moran’s *I* values of 0.29, 0.17, and 0.25, respectively; *p* < 0.05), indicating spatial clustering that passed the statistical significance test.

#### 3.2.2. County-Level Local Moran’s *I* Hotspots

**Figure 6:**
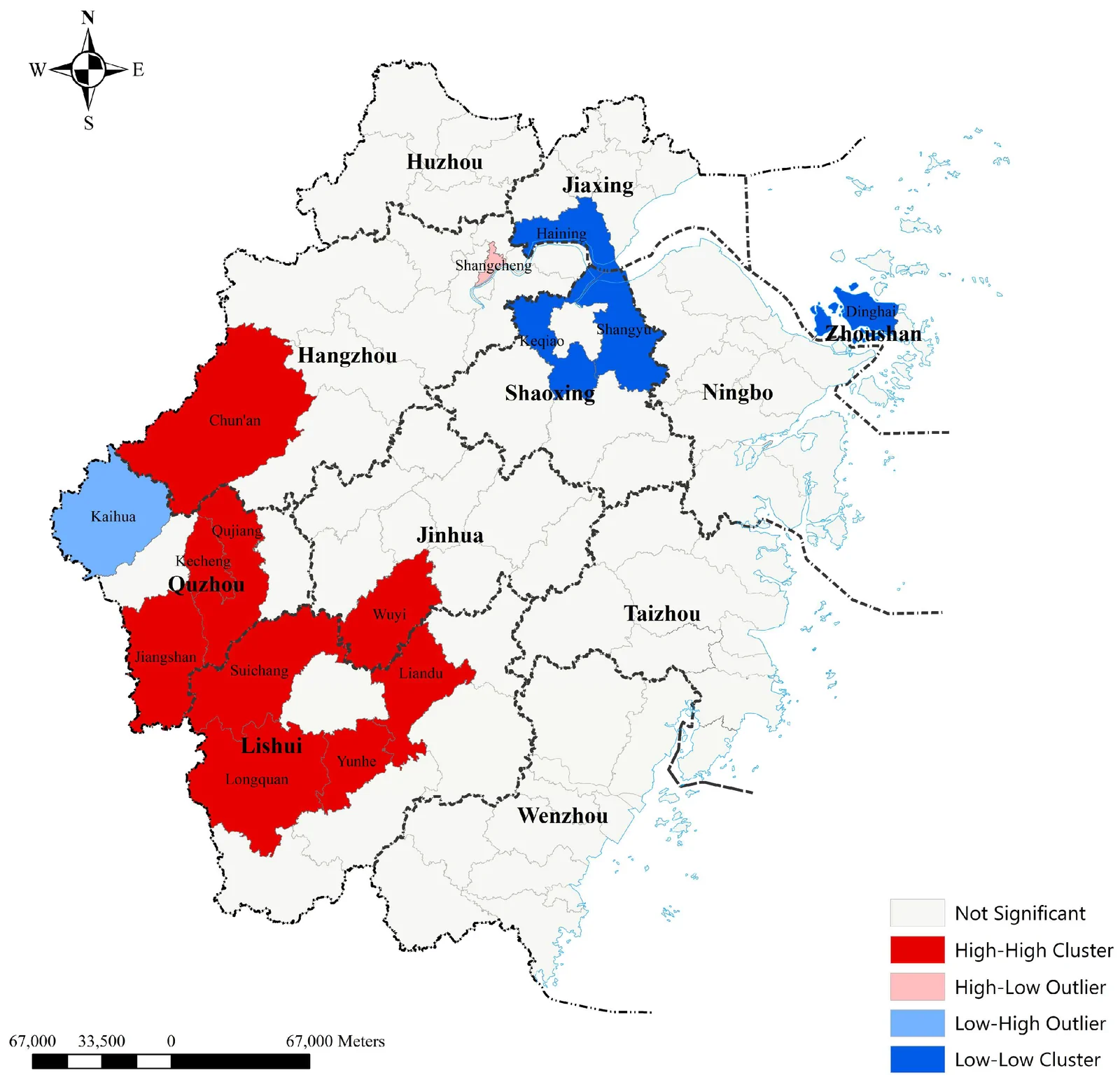
Spatial clustering analysis of county-level incidence in Zhejiang Province from 2018 to 2023.

Zhejiang Province has a total of 90 counties (county-level cities and districts). The local spatial autocorrelation of the average reported incidence from 2012 to 2023 is shown in Figure 6. In the figure, the high–high clustering areas mainly include Qujiang District, Suichang County, Chun’an County, Jiangshan City, Kecheng District, Longquan County, Yunhe County, Liandu District, and Wuyi County. These are the “hotspot” areas for the incidence of mushroom poisoning in Zhejiang Province from 2018 to 2023, primarily concentrated in the western part of Zhejiang. This region and its surrounding areas are all high-incidence areas for mushroom poisoning, exhibiting a high-value clustering pattern (*p* < 0.05). High–low outlier clusters were mainly found in Shangcheng District. The incidence of mushroom poisoning in this district is relatively high, but the incidence in its surrounding districts and counties is relatively low, exhibiting an anomalous distribution pattern of isolated high values. Low–high outlier clusters were found in Kaihua County. The incidence of mushroom poisoning in these counties and districts is relatively low, but the incidence in their surrounding counties and districts is relatively high. The low–low clustering areas mainly include Shangyu District, Keqiao District, Haining City, and Dinghai District, concentrated in northern Shaoxing and Haining City of Jiaxing in the Hangzhou Bay area of northern Zhejiang, as well as Dinghai District, Zhoushan City.

### 3.3. Spatiotemporal Clustering Characteristics

#### 3.3.1. Annual Spatiotemporal Clusters Detected by SaTScan, 2018–2023

To deeply investigate the spatiotemporal distribution patterns of mushroom poisoning in Zhejiang Province, this study employed SatScan v10.3.3 software to conduct a retrospective spatiotemporal scan analysis of surveillance data from 2018 to 2023. The results indicate that mushroom poisoning in Zhejiang Province exhibits a significantly non-random distribution across spatiotemporal dimensions, with pronounced clustering heterogeneity (*p* < 0.01).

Overall, the clustering intensity peaked in 2023, with an LLR of 114.49, significantly higher than in other years, suggesting that the clustering of cases within specific spatiotemporal units was most prominent in that year. Additionally, 2019 (LLR = 101.72) and 2018 (LLR = 91.88) also demonstrated strong clustering characteristics (Table 3).

**Figure 7:**
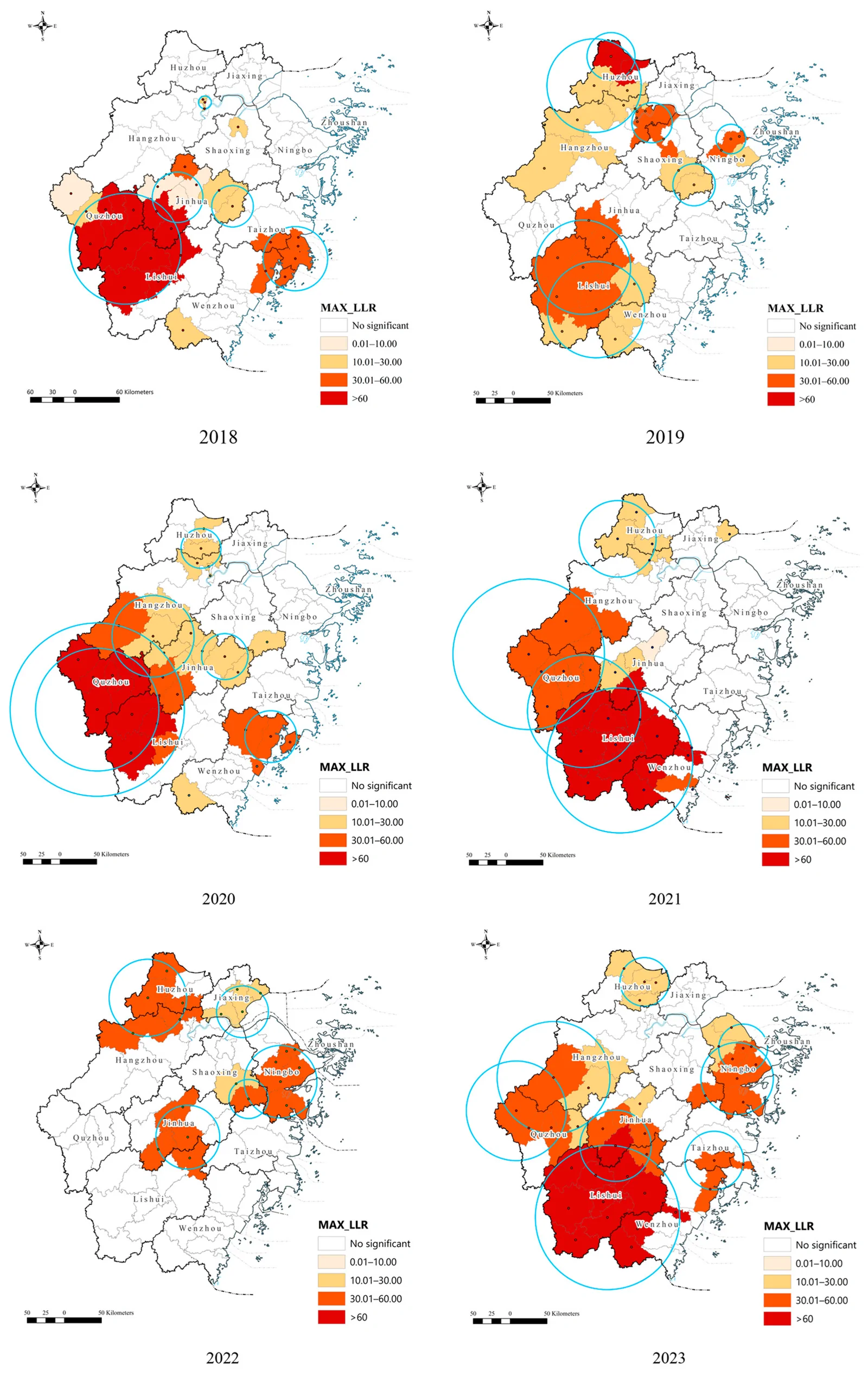
Visualization of retrospective case scanning in Zhejiang Province, 2018–2023. Blue circles delineate the overall high-risk spatiotemporal clusters detected by SaTScan.

From a spatial distribution perspective, poisoning incidents overall exhibited a pattern of frequent occurrences in the western region and fewer in the eastern region, with poisoning concentrated in mountainous areas. The most likely clusters over the years were consistently concentrated in western and southwestern Zhejiang’s mountainous counties and cities, primarily including Lishui, Quzhou, and western Jinhua (Figure 7). From a temporal distribution perspective, the clustering period was highly concentrated between June and October each year, closely aligning with the peak mushroom growth and foraging seasons in summer and autumn.

Taking 2023, the year with the highest clustering intensity, as an example, the most likely cluster comprised 12 districts and counties, including Lishui 331127 (LLR = 114.49, RR = 15.44), with a time window from August 4 to September 2 (Table 3). This cluster covers a wide area and exhibits a high relative risk, indicating that the mountainous regions of southwestern Zhejiang during late summer and early autumn are the core priority areas for mushroom poisoning prevention and control.

#### 3.3.2. County-Level Recurrence of Spatiotemporal Clusters and Ecological Context

To explore the spatial association between spatiotemporal clustering areas and ecological environmental characteristics, this study calculated the cumulative frequency of districts and counties identified as clustering areas (including the most likely cluster and secondary clusters) in each annual spatiotemporal scan from 2018 to 2023. All districts and counties were grouped by their cumulative frequency to compare differences in their environmental characteristics.

**Figure 8:**
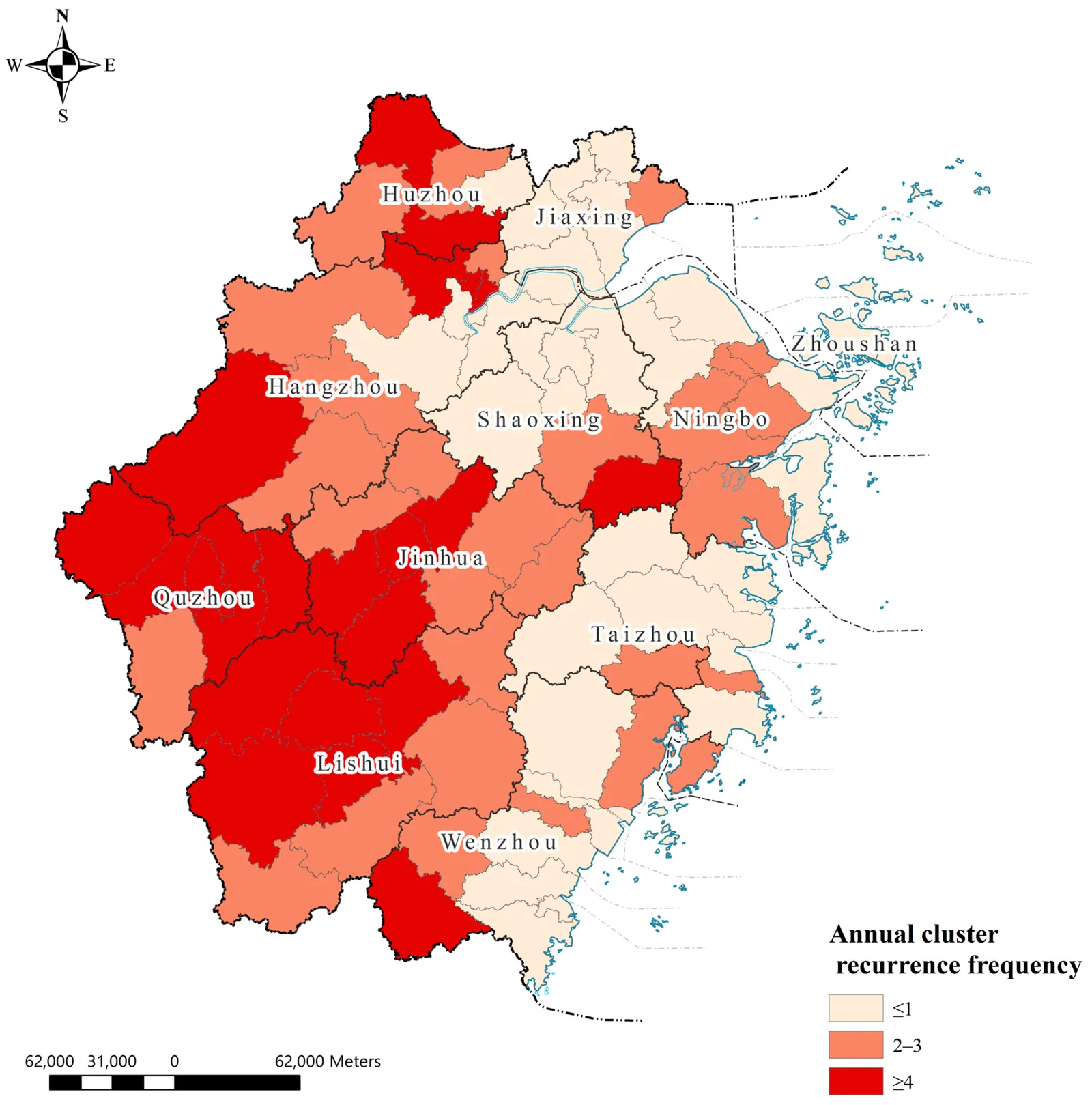
Annual cluster recurrence frequency of mushroom poisoning at the county level in Zhejiang Province, 2018–2023.

Figure 8 presents the spatial distribution of the cumulative clustering frequency of each district and county in Zhejiang Province over the 7-year scan period. High-frequency districts and counties (with the darkest color scale, ≥4 times) are mainly concentrated in the mountainous regions of western and southwestern Zhejiang, covering most districts and counties in Lishui, Quzhou and western Jinhua, forming a relatively continuous high-frequency clustering zone. In contrast, districts and counties in the eastern Zhejiang coastal areas and northern Zhejiang plains generally have low cumulative frequencies (with the lightest color scale, ≤1 time) and are only occasionally identified as clustering areas in individual years.

The mean values of environmental variables across the three groups are compared (Table 4). The results show that with the increase in clustering frequency, the mean NDVI rises gradually from 0.67 to 0.78, the mean forest coverage rate increases from 44.23% to 69.41%, and the average elevation rises from 98.81 m to 317.04 m. The high-frequency group outperforms the low-frequency group across all three environmental indicators, with a distinct gradient trend observed.

These descriptive results indicate that high-frequency districts and counties repeatedly identified as clustering areas have higher vegetation coverage, forest coverage and elevation, which is highly consistent with the geomorphic features of dense forests and high elevation in the mountainous regions of western Zhejiang. The consistency between this environmental gradient and clustering frequency suggests that the forest ecological environment in mountainous areas may provide favorable conditions for the growth of wild poisonous mushrooms and residents’ foraging exposure. Specifically, areas with better vegetation coverage, higher forest coverage and higher elevation show stronger suitability for mushroom growth, and correspondingly, residents in these areas face a higher risk of exposure to poisonous mushrooms.

However, the analysis in this study represents a descriptive comparison at the ecological level, and the causal relationship between the aforementioned environmental factors and poisonous mushroom poisoning cannot be definitively confirmed. Further multivariate analysis integrating meteorological factors, land use and behavioral exposure is needed in future studies.

## 4. Discussion

### 4.1. Spatiotemporal Patterns and Ecological Environmental Background of Mushroom Poisoning in Zhejiang Province

This study demonstrates that poisonous mushroom poisoning in Zhejiang Province presents distinct spatiotemporal clustering characteristics: temporally concentrated in summer and autumn (June to October), and spatially clustered mainly in the mountainous regions of western Zhejiang. Farmers constitute the majority of the affected population, and households are the primary locations of poisoning incidents. The main poisonous mushroom species involved include *Chlorophyllum molybdites* and related *Russula* groups, with gastroenteritis-type symptoms as the predominant clinical manifestation. Retrospective spatiotemporal scanning further identified high-risk clustering areas at the district and county level, among which the cluster in the Lishui area from August to September 2023 had the highest relative risk (RR = 15.44, LLR = 114.49).

The above clustering pattern may reflect the spatial features shaped by the combined effects of ecological environmental background and population exposure behavior. At the environmental level, districts and counties with recurrent clustering have higher forest coverage, vegetation greenness and average elevation, providing favorable ecological conditions for the growth and reproduction of wild mushrooms. However, environmental suitability only forms the geographical background of potential risk, while human behavior may be a critical factor mediating the conversion of potential exposure into actual poisoning incidents. The epidemiological features in this study—the high proportion of farmers and the predominance of household settings—are consistent with the long-standing behavioral pattern of wild mushroom foraging and consumption in rural areas. Therefore, the spatial clustering of poisonous mushroom poisoning in the mountainous regions of western Zhejiang may result from the joint effects of natural ecological conditions and residents’ exposure behavior.

### 4.2. Differences in Species Characteristics and Their Implications for Poisoning Prevention and Control

Compared with other high-incidence provinces in China, the mushroom poisoning species spectrum in Zhejiang Province exhibits significant regional specificity. In Yunnan, Sichuan, and other regions, highly toxic *Amanita* species (e.g., *Amanita exitialis*, *Amanita fuliginea*) are dominant, with poisoning case fatality rates reaching over 16% [24,25]. In contrast, the dominant poisonous mushroom species reported in Zhejiang Province mainly include *Chlorophyllum molybdites* and related *Russula* groups traditionally identified as *Russula japonica*. Their toxic manifestations are predominantly of the gastroenteritis type, with few fatal cases. This regional difference may be associated with variations in the species composition and ecological characteristics of poisonous mushrooms across different regions.

Differences in ecological characteristics among different poisonous mushroom species may explain their distinct distribution patterns. *Amanita* and *Russula* species are mostly ectomycorrhizal fungi, forming symbiotic relationships with specific tree species such as Fagaceae and Pinaceae, and are mostly distributed in primary forests or well-preserved natural vegetation areas. In contrast, *Chlorophyllum molybdites* is a saprophytic fungus that thrives in disturbed anthropogenic habitats rich in organic matter, such as rural courtyards, vegetable plots, roadsides, and garbage dumps and other areas with frequent human activities. Therefore, the high-risk areas identified in the mountainous western Zhejiang may reflect the combined effects of forest ecosystems and rural residential environments. Forests provide suitable habitats for ectomycorrhizal fungi (e.g., *Russula*), whereas rural residential environments offer favorable growth conditions for saprophytic opportunistic fungi (e.g., *Chlorophyllum molybdites*). The superposition of these two factors forms the high-incidence pattern in this region. Mushroom identification primarily relies on expert assessment of external morphology, microscopic characteristics, and DNA molecular markers [26,27]; it is difficult for the general public to distinguish edible mushrooms from toxic ones, increasing the likelihood of accidental ingestion. The relatively high fatality risk in Yunnan is primarily associated with exposure to highly toxic species such as lethal *Amanita* mushrooms. In contrast, Zhejiang is characterized by a higher incidence of poisoning cases, most of which involve gastrointestinal toxicity.

It should be noted that there remains a certain degree of uncertainty regarding the species identification of *Russula* taxa involved in this study. Recent molecular phylogenetic studies have revealed that specimens previously identified as *Russula japonica* across China may actually represent multiple distinct species, including four newly described species within the subgenus *Brevipedum* of *Russula*. Since this study relies on retrospective surveillance data without DNA sequence verification, molecular re-identification of all historical specimens cannot be performed. Future research integrating morphological identification and molecular biological approaches is required to further clarify the species diversity and toxic characteristics of poisonous *Russula* species in Zhejiang Province.

Given the above ecological and species discrepancies, the identification of this niche mechanism implies that Zhejiang’s prevention and control strategy cannot simply replicate Yunnan’s goal of treating high-fatality poisoning cases but must shift towards reducing the frequency of poisoning incidents. This involves identifying the growth patterns of *Chlorophyllum molybdites* around residential areas to reduce exposure opportunities at the source.

### 4.3. Development of the Surveillance System and Prevention and Control Strategies

It is noteworthy that the number of mushroom poisoning cases in Zhejiang Province has been continuously rising since 2016, a trend consistent with reports from Hubei, Guizhou, Guangxi, and other provinces [28,29,30]. However, the sharp surge in reported cases should not be simply interpreted as a genuine rise in population-level incidence; instead, it may reflect the combined systematic effects of expanded surveillance coverage and reduced underreporting rates. During the study period, Zhejiang’s foodborne disease surveillance network underwent continuous expansion. From 2012 to 2015, the number of medical institutions participating in surveillance in Zhejiang Province gradually increased from an initial 10 to 101. Starting in 2016, all 340 secondary-level and above medical institutions across all counties (cities, districts) in Zhejiang Province fully implemented foodborne disease surveillance. From 2018 onward, surveillance institutions were expanded to include all medical institutions in the province that treat foodborne disease cases, including community health service centers and township health centers. Meanwhile, since 2018, the Provincial Center for Disease Control and Prevention has organized special surveillance for mushroom poisoning and established an expert identification group and a specimen submission mechanism, significantly improving the ability of primary-level medical staff to identify and report mushroom poisoning.

Therefore, the increase in reported case numbers largely reflects a reduction in underreporting of mushroom poisoning incidents rather than a simple increase in poisoning events. Some mild poisoning cases may not have sought medical attention, resulting in reported poisoning incidents being lower than the actual number. This institutional explanation suggests that when interpreting temporal trends, it is necessary to distinguish between real changes in disease incidence and changes in the surveillance system. Future early warning models should consider incorporating surveillance coverage as a correction factor.

Compared with international experience, China’s approach to preventing and controlling mushroom poisoning also differs. European and American countries often restrict the commercial harvesting and sale of wild mushrooms through legislation and establish professional identification networks to support clinical diagnosis [31,32]. In contrast, China currently relies primarily on health education, depending on the public’s increased awareness to avoid consuming wild mushrooms. However, practices in Zhejiang and other regions show that relying solely on public communication is insufficient to eradicate long-established foraging habits, especially in rural areas, where traditional beliefs that wild mushrooms have high nutritional value reduce the effectiveness of public communication.

The high-risk spatiotemporal clusters identified through retrospective spatiotemporal scanning in this study (e.g., Lishui area in August–September 2023, RR = 15.44) possess substantial practical value for public health practice.

First, for high-risk counties in western Zhejiang and the high-incidence period from June to October, historical clustering patterns can be used to disseminate identification guides for locally prevalent poisonous mushroom species through dialect radio broadcasts and village-level WeChat groups before the annual epidemic season to raise residents’ risk awareness.

Second, based on the annual spatiotemporal clustering patterns, grassroots medical resource allocation can be optimized in advance to prepare for sudden surges in reported cases.

Furthermore, spatiotemporal scan methods can provide technical support for establishing foodborne disease surveillance and early warning mechanisms based on historical risk patterns.

### 4.4. Limitations

This study still has certain limitations. First, the spatial clustering analysis is primarily based on county-level administrative units, with a relatively coarse spatial resolution that may mask local heterogeneity at the village scale. Future research could integrate more refined geographic information of reported cases and high-resolution remote sensing data (e.g., NDVI, land surface temperature, land use types) to further explore the environmental exposure context of poisonous mushrooms.

Second, this study has not yet been refined to further incorporate meteorological variables (e.g., precipitation, temperature, humidity), although the occurrence of mushroom fruiting bodies is closely related to rainfall. Subsequent studies could construct dynamic correlation models between the lag effect of rainfall and poisoning peaks—for example, by analyzing the temporal association between rainfall events and poisoning peaks—to further refine the timeliness of spatiotemporal predictions.

In addition, this study is retrospective in design, and the clustering patterns identified reflect historical regularities. The absence of an appropriate comparison population in the surveillance dataset limited further quantitative assessment of risk factors associated with mushroom poisoning occurrence. Future prospective cohort or quasi-experimental studies are needed to validate the effectiveness of intervention measures formulated based on these patterns in actual prevention and control. Future research may integrate prospective surveillance, population-based epidemiological data, and intervention evaluation studies to further investigate the determinants of mushroom poisoning and verify the practical effectiveness of precision prevention and control strategies derived from spatiotemporal risk identification results.

## 5. Conclusions

Based on mushroom poisoning surveillance data from Zhejiang Province from 2012 to 2023, this study systematically revealed the spatiotemporal distribution characteristics and clustering patterns of poisoning incidents by employing descriptive epidemiology, spatial autocorrelation analysis, and spatiotemporal scan statistics, confirming that mushroom poisoning in Zhejiang Province exhibits stable and identifiable spatiotemporal clustering characteristics.

The above results indicate that retrospective analysis based on spatiotemporal scanning can effectively identify high-risk spatiotemporal clusters, providing a basis for shifting mushroom poisoning research and intervention from passive case collection and reporting to targeted intervention. Future prevention and control efforts should focus on the following aspects: for hotspot counties in western Zhejiang and the high-incidence period from June to October, utilize historical clustering patterns to conduct targeted and timed education campaigns, and strengthen the identification and risk awareness of dominant species such as *Chlorophyllum molybdites*; integrate the spatiotemporal scanning model into the surveillance platform to enable automatic identification of high-risk spatiotemporal clusters and internal risk alerts; promote multi-sectoral collaboration among disease control, healthcare, and community agencies; and conduct active surveillance and follow-up in hotspot areas during the high-incidence season. Subsequent research can introduce meteorological and environmental variables to optimize the model, and validate prevention and control effectiveness through prospective intervention experiments, providing scalable experience for similar regions nationwide.

## Figures and Tables

**Table 1:** Statistics of Mushroom Poisoning Cases by City in Zhejiang Province.

City	2012	2013	2014	2015	2016	2017	2018	2019	2020	2021	2022	2023
Hangzhou	0	0	1	0	8	14	17	77	32	26	37	54
Ning-bo	0	0	0	4	26	14	10	41	18	10	37	88
Wenzhou	0	0	0	6	9	16	20	16	46	72	16	104
Jiaxing	0	0	0	1	0	3	6	7	9	13	23	30
Huzhou	0	0	1	0	0	6	14	49	15	14	27	60
Shao-xing	0	0	5	4	2	0	15	33	11	5	21	17
Jin-hua	0	0	5	2	23	18	57	19	54	23	37	124
Quzhou	6	0	8	9	6	13	56	14	54	28	8	108
Zhou-shan	0	0	0	0	1	0	0	0	0	1	0	2
Taizhou	0	0	1	0	7	9	15	1	6	9	15	96
Lishui	0	0	15	3	6	14	36	32	24	27	12	62
Total	6	0	36	29	88	107	246	289	269	228	233	745

**Table 2:** Results of clustering analysis of mushroom poisoning cases in Zhejiang Province, 2018–2023.

Statistic	2018	2019	2020	2021	2022	2023
Moran’s *I*	0.21	0.22	0.17	0.25	0.07	0.12
*p*-value	0.06	0.47	0.04 *	0.04 *	0.83	0.13

*: *p* < 0.05.

**Table 3:** Characteristics of the most significant annual clusters of mushroom poisoning in Zhejiang Province from 2018 to 2023.

Year	Center Coordinates/Radius	Cluster Area (Example/Core Area)	Cluster Time (Start and End)	Number of Districts and Counties	RR Value	LLR Value
2018	(28.52° N, 119.08° E)/74.83 km	331123 (Lishui) and 11 other counties	4 August 2018–2 September 2018	12	26.85	91.88
2019	(30.98° N, 119.81° E)/32.24 km	330522, 330502 (Huzhou)	3 September 2019–2 October 2019	2	61.93	101.72
2020	(28.58° N, 118.60° E)/83.59 km	330881 (Quzhou) and 8 other counties	3 September 2020–2 October 2020	9	28.66	83.59
2021	(27.92° N, 119.60° E)/98.51 km	331127 (Lishui) and 12 other counties	4 August 2021–2 September 2021	13	27.42	88.08
2022	(29.60° N, 121.41° E)/48.87 km	330213 (Ningbo) and 6 other counties	3 September 2022–2 October 2022	7	19.80	49.60
2023	(27.92° N, 119.60° E)/97.40 km	331127 (Lishui) and 11 other counties	4 August 2023–2 September 2023	12	15.44	114.49

Complete year-by-year scanning results are detailed in Appendix A Table A1, Table A2, Table A3, Table A4, Table A5 and Table A6.

**Table 4:** Grouped comparison of environmental characteristics by annual cluster recurrence frequency of mushroom poisoning at the county level in Zhejiang Province, 2018–2023.

Annual Cluster Recurrence Frequency	NDVI	Forest Coverage Rate (%)	Mean Elevation (m)
≤1	0.67	44.23	98.81
2–3	0.77	64.43	268.15
≥4	0.78	69.41	317.04

## Data Availability

The datasets presented in this article are not readily available because the data are sourced from the Zhejiang Province Foodborne Disease Surveillance Reporting System, which is not currently open to the public. Requests to access the datasets should be directed to the corresponding authors.

## Appendix A

**Table A1:** The most significant cluster characteristics of mushroom poisoning in Zhejiang Province in 2018.

Time	Cluster Area	Cluster Duration	Number of Districts	RR Value	LLR Value	*p*-Value
2018	331123 and 11 other counties	4 August 2018–2 September 2018	12	26.85	91.88	<0.001
2018	330726	3 September 2018–2 October 2018	1	64.07	31.55	<0.001
2018	331081 and 5 other counties	4 August 2018–2 September 2018	6	11.66	30.13	<0.001
2018	330602	4 August 2018–2 September 2018	1	27.76	21.08	<0.001
2018	330822	5 June 2018–4 July 2018	1	52.58	20.77	<0.001
2018	330103 and 2 other counties	3 September 2018–2 October 2018	3	20.95	18.66	<0.001
2018	330727	5 June 2018–4 July 2018	1	58.92	15.42	<0.001
2018	330329	5 June 2018–4 July 2018	1	33.61	12.67	0.00097
2018	330726	6 May 2018–4 June 2018	1	31.37	12.34	0.0013
2018	330727, 330783	4 August 2018–2 September 2018	2	14.28	10.31	0.0095
2018	330703 and 3 other counties	5 June 2018–4 July 2018	4	8.22	9.73	0.015
2018	330824	7 March 2018–5 April 2018	1	28.09	9.45	0.021

**Table A2:** The most significant cluster characteristics of mushroom poisoning in Zhejiang Province in 2019.

Time	Cluster Area	Cluster Duration	Number of Districts	RR Value	LLR Value	*p*-Value
2019	330522, 330502	3 September 2019–2 October 2019	2	61.93	101.72	<0.001
2019	331124 and 7 other counties	5 June 2019–4 July 2019	8	24.48	57.07	<0.001
2019	330205 and 2 other counties	5 July 2019–3 August 2019	3	32.17	42.05	<0.001
2019	330102	5 July 2019–3 August 2019	1	76.34	36.62	<0.001
2019	330109 and 5 other counties	3 September 2019–2 October 2019	6	13.60	36.29	<0.001
2019	330624, 330683	5 June 2019–4 July 2019	2	24.88	29.03	<0.001
2019	330122	3 September 2019–2 October 2019	1	36.48	18.29	<0.001
2019	330127	4 August 2019–2 September 2019	1	33.30	17.67	<0.001
2019	331127 and 8 other counties	3 September 2019–2 October 2019	9	9.45	16.02	<0.001
2019	330523 and 6 other counties	5 June 2019–4 July 2019	7	6.70	13.45	0.00020
2019	330212	3 September 2019–2 October 2019	1	15.92	12.74	0.00043
2019	330328	5 June 2019–4 July 2019	1	26.56	11.54	0.0015

**Table A3:** The most significant cluster characteristics of mushroom poisoning in Zhejiang Province in 2020.

Time	Cluster Area	Cluster Duration	Number of Districts	RR Value	LLR Value	*p*-Value
2020	330881 and 8 other counties	3 September 2020–2 October 2020	9	28.66	83.59	<0.001
2020	330881 and 12 other counties	5 June 2020–4 July 2020	13	15.76	53.05	<0.001
2020	330382 and 2 other counties	3 September 2020–2 October 2020	3	18.97	41.13	<0.001
2020	330303	6 June 2020–4 July 2020	1	69.83	32.42	<0.001
2020	330624	5 June 2020–4 July 2020	1	49.42	26.14	<0.001
2020	330521 and 3 other counties	5 June 2020–4 July 2020	4	13.40	24.68	<0.001
2020	330783 and 2 other counties	5 June 2020–4 July 2020	3	11.14	13.38	0.00035
2020	330102	3 September 2020–2 October 2020	1	36.56	13.09	0.00047
2020	330182 and 5 other counties	3 October 2020–1 November 2020	6	8.54	12.47	0.00087
2020	330329	3 October 2020–1 November 2020	1	31.42	12.35	0.00097
2020	330782	4 August 2020–2 September 2020	1	16.54	11.14	0.0033

**Table A4:** The most significant cluster characteristics of mushroom poisoning in Zhejiang Province in 2021.

Time	Cluster Area	Cluster Duration	Number of Districts	RR Value	LLR Value	*p*-Value
2021	331127 and 12 other counties	4 August 2021–2 September 2021	13	27.42	88.08	<0.001
2021	330326	3 October 2021–1 November 2021	1	42.56	35.70	<0.001
2021	330824 and 7 other counties	4 August 2021–2 September 2021	8	15.03	31.33	<0.001
2021	330703	4 August 2021–2 September 2021	1	56.45	21.25	<0.001
2021	331102	6 April 2021–5 May 2021	1	39.11	16.08	<0.001
2021	330482	4 August 2021–2 September 2021	1	32.52	15.00	<0.001
2021	330523 and 4 other counties	5 July 2021–3 August 2021	5	9.96	13.81	0.00022
2021	331123 and 10 other counties	3 October 2021–1 November 2021	11	7.51	12.44	0.00088
2021	330782	5 June 2021–4 July 2021	1	15.97	9.12	0.030

**Table A5:** The most significant cluster characteristics of mushroom poisoning in Zhejiang Province in 2022.

Time	Cluster Area	Cluster Duration	Number of Districts	RR Value	LLR Value	*p*-Value
2022	330213 and 6 other counties	3 September 2022–2 October 2022	7	19.80	49.60	<0.001
2022	330784 and 4 other counties	5 June 2022–4 July 2022	5	19.39	35.63	<0.001
2022	330523 and 5 other counties	4 August 2022–2 September 2022	6	15.09	31.41	<0.001
2022	330424 and 3 other counties	4 August 2022–2 September 2022	4	16.82	24.14	<0.001
2022	330624, 330683	5 June 2022–4 July 2022	2	16.88	13.10	0.00042

**Table A6:** The most significant cluster characteristics of mushroom poisoning in Zhejiang Province in 2023.

Time	Cluster Area	Cluster Duration	Number of Districts	RR Value	LLR Value	*p*-Value
2023	331127 and 11 other counties	4 August 2023–2 September 2023	12	15.44	114.49	<0.001
2023	330213 and 6 other counties	5 July 2023–3 August 2023	7	10.09	59.61	<0.001
2023	330824 and 4 other counties	3 September 2023–2 October 2023	5	14.38	56.62	<0.001
2023	330723 and 6 other counties	5 June 2023–4 July 2023	7	10.40	52.44	<0.001
2023	331003 and 2 other counties	4 August 2023–2 September 2023	3	9.39	34.64	<0.001
2023	330782	4 August 2023–2 September 2023	1	15.29	28.53	<0.001
2023	330522	3 September 2023–2 October 2023	1	18.67	27.60	<0.001
2023	330502 and 3 other counties	5 June 2023–4 July 2023	4	7.32	21.24	<0.001
2023	330127 and 7 other counties	5 July 2023–3 August 2023	7	6.18	20.43	<0.001
2023	330205 and 5 other counties	3 September 2023–2 October 2023	6	4.56	16.02	<0.001
2023	330329	5 June 2023–4 July 2023	1	16.01	12.82	0.00075
2023	331024, 330727	5 June 2023–4 July 2023	2	8.08	8.47	0.057
